# Assessment of Response to Chemotherapy in Pancreatic Cancer with Liver Metastasis: CT Texture as a Predictive Biomarker

**DOI:** 10.3390/diagnostics11122252

**Published:** 2021-12-01

**Authors:** Sihang Cheng, Zhengyu Jin, Huadan Xue

**Affiliations:** Department of Radiology, Peking Union Medical College Hospital, Chinese Academy of Medical Sciences, Beijing 100730, China; chengsihangscu@foxmail.com (S.C.); zhengyu_jin@163.com (Z.J.)

**Keywords:** pancreatic cancer, liver metastasis, chemotherapy, texture analysis, response

## Abstract

In this paper, we assess changes in CT texture of metastatic liver lesions after treatment with chemotherapy in patients with pancreatic cancer and determine if texture parameters correlate with measured time to progression (TTP). This retrospective study included 110 patients with pancreatic cancer with liver metastasis, and mean, entropy, kurtosis, skewness, mean of positive pixels, and standard deviation (SD) values were extracted during texture analysis. Response assessment was also obtained by using RECIST 1.1, Choi and modified Choi criteria, respectively. The correlation of texture parameters and existing assessment criteria with TTP were evaluated using Kaplan-Meier and Cox regression analyses in the training cohort. Kaplan-Meier curves of the proportion of patients without disease progression were significantly different for several texture parameters, and were better than those for RECIST 1.1-, Choi-, and modified Choi-defined response (*p* < 0.05 vs. *p* = 0.398, *p* = 0.142, and *p* = 0.536, respectively). Cox regression analysis showed that percentage change in SD was an independent predictor of TTP (*p* = 0.016) and confirmed in the validation cohort (*p* = 0.019). In conclusion, CT texture parameters have the potential to become predictive imaging biomarkers for response evaluation in pancreatic cancer with liver metastasis.

## 1. Introduction

Pancreatic cancer is among the top cancers with high mortality affecting over 200,000 deaths every year, worldwide [1,2]. Unfortunately, a large proportion of patients are identified at an advanced stage with poor prognosis, especially for metastatic pancreatic cancer, for which the liver is the most common site, accounting for 37–41.9% of the initially diagnosed cases, with a 5-year overall survival (OS) rate of 2%, and a median life expectancy of less than 1 year [3,4]. FOLFIRINOX and gemcitabine-based chemotherapy regimens are common treatments for metastatic pancreatic cancer. It was observed that FOLFIRINOX was superior to gemcitabine alone in progression-free survival (PFS), response, and OS in the treatment of metastatic pancreatic cancer, and another phase 3 trial confirmed the superiority of gemcitabine plus nanoparticle albumin-bound paclitaxel (nab-paclitaxel) as well [5,6]. Assessment of treatment response to chemotherapy is important, especially for the non-responders, which might provide these patients opportunities to find more appropriate treatment plans in time.

The response evaluation criteria in solid tumors (RECIST) as a frequently used tool for the assessment of tumor response only takes size change of the lesions into account [7]. Choi et al. developed comprehensive criteria incorporating changes in both tumor size and attenuation, which defines partial response (PR) as a decrease of >10% in tumor size or a decrease of >15% in tumor attenuation. These criteria define progressive disease (PD) as a tumor size increase of >10% without meeting the PR criteria [8]. The definition of PR according to the modified Choi criteria is a 15% reduction in enhancement and a 10% reduction in size [9]. No studies have been conducted to assess the response to chemotherapy in pancreatic cancer with liver metastasis using these criteria.

Analysis of tumor lesion heterogeneity will reveal vital information concerning response to treatment [10,11]. CT texture analysis is an emerging technique used to process images and hence help to characterize lesion heterogeneity. This technique analyzes the relationship and distribution of pixel gray levels within a lesion and reveals spatial variations among individual gray patterns or levels [10,12,13]. Extracted features include kurtosis, skewness, mean of positive pixels (MPP), entropy, standard deviation (SD), and mean gray-level intensity (MI) [14]. It has been proved that CT texture analysis played an important role in the assessment of tumor response to various treatments and had the potential to establish a more precise assessment criteria for response evaluation [15,16,17,18]. However, texture analysis as a non-invasive tool for the assessment of chemotherapy treatment response in pancreatic cancer with liver metastasis has not been investigated before.

In this study, we assessed alterations in CT texture of metastatic liver lesions following gemcitabine-based chemotherapy in pancreatic cancer patients and then compared the effectiveness of this approach with existing assessment criteria in evaluating treatment response and determining time to progression (TTP).

## 2. Materials and Methods

### 2.1. Study Population

Prior to the study, approval of this study was waived by the institutional review board of Peking Union Medical College Hospital for its retrospective nature. Patients with pancreatic cancer with liver metastasis undergoing chemotherapy between September 2014 and October 2018 were identified from our institutional electronic medical database. Inclusion criteria: (a) They were pathologically diagnosed to be pancreatic cancer with liver metastasis; (b) they were chemotherapy naïve and received gemcitabine-based chemotherapy (gemcitabine plus an oral fluoropyrimidine anticancer agent, S-1) as first-line treatment; (c) baseline contrast-enhanced CT had been done in 2 weeks prior to treatment initiation; (d) contrast-enhanced CT had been done following treatment to monitor response. A total of 168 patients met the inclusion criteria. Patients were excluded if they had previously undergone radiofrequency or microwave ablation of the liver metastatic lesions (*n* = 17), had infiltrative HCC without any accurately delineable lesion (*n* = 20), died during the first 15 days after treatment initiation (*n* = 3), and were without a regular documented follow-up after chemotherapy initiation or until disease progression (*n* = 18).

### 2.2. Follow-Up and Endpoints

The follow-up (radiological, clinical, and biological) was carried out every 2 months as outlined by the institutional protocol. During follow-up, radiological assessment was performed with contrast-enhanced CT scans of the pelvis, abdomen, and thorax. TTP was defined as the time from the start of chemotherapy to radiologic progression, which was the chosen end point detailed by RECIST 1.1. Participants who did not exhibit radiologic progression at the end of the follow-up were censored.

### 2.3. Protocol of CT Examination

All images were acquired in the Department of Radiology at our hospital. All the scans were done on 128-detector CT scanners (Siemens SOMATOM Definition Flash, Siemens Healthcare, Forchheim, Germany). The scanning parameters were as follows: tube voltage, 120 kVp; tube current, 150 mAs (thorax) or 200 mAs (abdomen and pelvis) with dose modulation; gantry rotation time, 0.5 s; table increment 46.8 mm per rotation; matrix 512 × 512. Images were routinely reconstructed with 5.0 mm slice thickness and 5.0 mm intervals. Non-ionic contrast media (Ultravist, 370 mg of iodine per milliliter, Schering, Berlin, Germany) were injected with 1.5 mL per kilogram of body weight at a rate of 3.0 mL/s using an automatic power injector. Bolus tracking was applied, and the thorax and upper abdomen (from the supraclavicular fossa to the iliac crest) were imaged in the arterial phase of enhancement (25-s delay), and the abdomen and pelvis (from the dome of the diaphragm to the pubis) were imaged in the portal venous phase of enhancement (70-s delay), according to the protocol in our institution.

### 2.4. Texture Analysis

**Figure 1:**
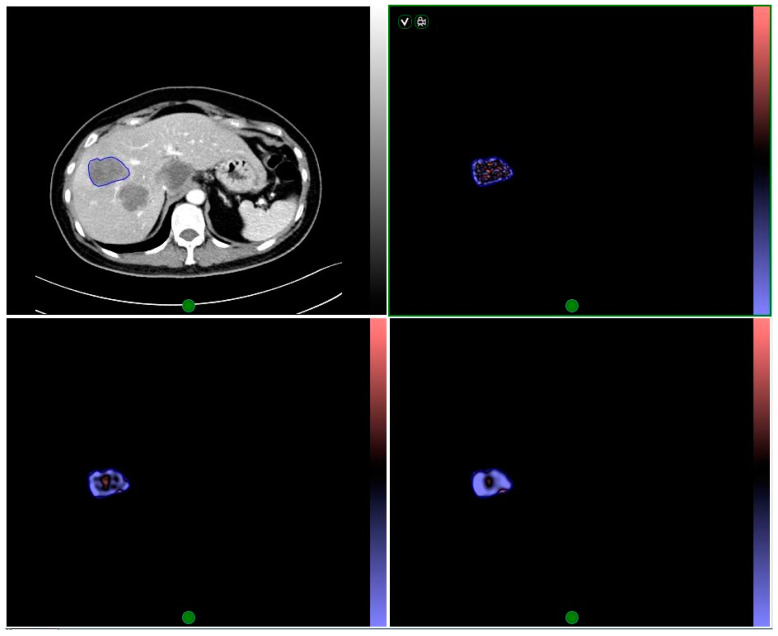
Illustration of lesion delineation, and image filtration at fine, medium, and coarse texture scales.

All images were analyzed by two radiologists (20 years and 5 years of abdominal imaging experience, respectively) who were blinded to the clinical outcome of the participants. Measurements were made on metastatic liver lesions according to the protocol provided by previous study [10], and tumor dimension and attenuation were measured using an image viewing software (Centricity, GE Medical Systems, Milwaukee, WI, USA). Texture analysis was carried out on the lesions using a commercial software (TexRAD, TexRAD Ltd., Cambridge, UK) by drawing a region of interest (ROI) around the peripheral margin of the lesions on CT images showing the largest area of the metastatic lesions. Various texture parameters were quantified prior and post to the application of spatial band-pass filters. Filter values on a scale of 0 to 6 were adjusted to select an appropriate scale. Where 0 = absence of filtration (spatial scaling factor (SSF) = 0), 2 = fine texture, 3 and 4 indicate different degrees of medium texture and 5 and 6 indicate different degrees of coarse texture. This analysis yielded derived CT texture images showing imaging characteristics at various spatial scales in the ROI (Figure 1).

### 2.5. Statistical Analysis

Quantitative data were shown as mean ± SD or medians. Categorical variables were presented as percentages. Mann-Whitney U test, Fisher exact, or Chi-square test were utilized appropriately to compare proportions and ranks of variables between training and validation cohorts. Texture parameters extracted from liver metastatic sites at baseline CT and at CT after two cycles of treatment, and the percentage change from the baseline value were all statistically analyzed. The association between texture parameters, RECIST 1.1, Choi, and modified Choi response criteria after two cycles of treatment and measured TTP were evaluated with the Kaplan-Meier analysis. Receiver operating characteristic (ROC) analysis was performed to identify threshold values for texture parameters. Kaplan-Meier analysis of participants with values below or above the thresholds were done to show the proportion of patients who did not have disease progression at any given time. Nonparametric log-rank test was performed to examine the differences between Kaplan-Meier curves for texture parameters, RECIST 1.1, Choi, and modified Choi criteria. Independent predictors of TTP among clinical and texture parameters were identified by performing multivariate analysis. All statistical analysis was done on SPSS 22.0 (IBM, New York, NY, USA). *p* < 0.05 indicated a significant difference, however, when facing multiple comparisons, a stepwise Holm–Bonferroni procedure was performed to reduce the potential for type I errors [19].

## 3. Results

In this study, we finally analyzed 110 pancreatic cancer patients with liver metastasis. These patients were randomly divided into two cohorts with 60 participants (mean age of 61.1 ± 8.6 years; range, 42–76 years) constituted a training cohort; while 50 participants (mean age, 56.1 ± 10.3 years; range, 31–73 years) constituted the validation cohort. The demographics and clinical features did not show significant difference between the validation and training cohorts (Table 1). In the training cohort, 10% of the patients (6/60) exhibited more than three liver target lesions, while in the validation cohort, 24% of patients (12/50) had >three target lesions. The initial treatment responses in the training cohort were shown in Table 2, categorized using RECIST 1.1, Choi, and modified Choi criteria. Follow-up imaging revealed that 42 of 60 patients in the training cohort and 32 of 50 patients in the validation cohort showed progression at the end of the follow-up. The median TTP was 178 (range, 36–1441) days in the validation cohort and 241 (range, 43–1164) days in the training cohort.

The median values with their ranges for texture parameters for absolute scale values at baseline and after two treatment cycles, as well as corresponding percentage change, were shown in Table 3.

**Figure 2:**
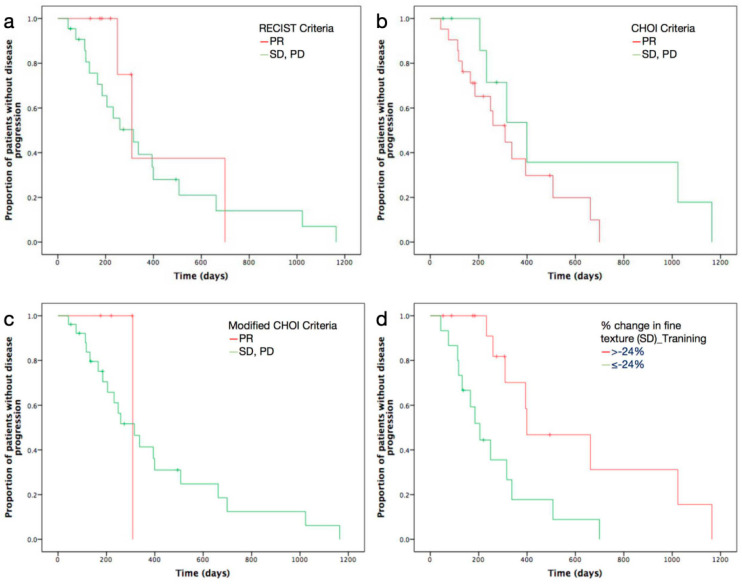
Kaplan-Meier curves show proportion of patients without disease progression for RECIST 1.1 (**a**), Choi (**b**), and modified Choi criteria (**c**) and percentage change in standard deviation (SD) (**d**).

Predictive ability of texture parameters at different SSF were shown in Table 4, Table 5 and Table 6. With fine texture (SSF = 2), baseline MI and percentage change in both SD and kurtosis, were found to be significantly correlated with TTP. With medium texture (SSF = 4), baseline kurtosis, and percentage change in MPP, were found to be significant predictors. Baseline kurtosis at SSF3, as well as percentage changes in entropy and MPP at SSF5, were also found to be significantly associated with TTP. Kaplan-Meier analysis of the proportion of patients without disease progression revealed significant differences for the above-mentioned texture parameters, which were better than those for RECIST 1.1, Choi-, and modified Choi-defined responses after chemotherapy (*p* value < 0.05 vs. *p* value = 0.398, *p* value = 0.142, and *p* value = 0.536, respectively) (Figure 2).

After the performance of multivariate Cox regression analysis, baseline MI at fine texture scale (SSF2, *p* value = 0.028), baseline kurtosis at medium texture scales (SSF3, *p* value = 0.032; SSF4, *p* value = 0.049), percentage change in kurtosis and SD at fine texture scale (SSF2, *p* value = 0.033 and *p* value = 0.016, respectively), and percentage change in MPP at medium texture scale (SSF4, *p* value = 0.016) were found to be independent prognostic factors for TTP (Table 7).

**Figure 3:**
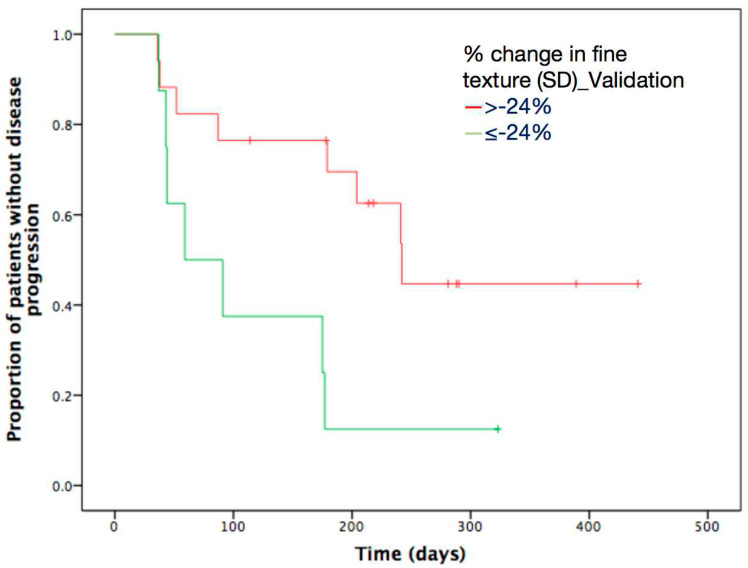
Kaplan-Meier survival curves according to percentage change in SD at fine (SSF2) texture scale in validation cohorts. Patients with percentage change in SD below optimal thresholds of −0.24 at fine texture scales showed significantly poorer survival in validation cohorts. TTP = time to progression.

However, these initially obtained results from training cohort needed further validation in an independent cohort. Optimal thresholds of above-mentioned independent texture parameters were acquired using ROC, and they were used to dichotomize texture parameters in the validation cohort. After application of Kaplan-Meier analysis, percentage change in SD at fine (SSF2) texture scale was confirmed as the only significant predictor of TTP (*p* value = 0.019) (Table 8). Percentage change in SD exceeding −24% at SSF2 was significantly associated with longer TTP following gemcitabine-based chemotherapy (Figure 3).

## 4. Discussion

In this study, we found that texture features of liver metastatic lesions on contrast-enhanced CT images could accurately reflect treatment response and predict TTP in pancreatic cancer patients treated with chemotherapy, and these texture parameters showed great advantages over traditional assessment criteria. Importantly, we demonstrated that the percentage change in SD at fine texture scale had the ability to serve as an independent predictive biomarker, and its correlated optimal threshold value was validated in an independent cohort of patients.

RECIST 1.1 as a commonly used imaging-defined response assessment tool, has been widely used in the evaluation of various cancers. However, accurate assessment of clinical benefits from chemotherapy using RECIST 1.1 was constrained by the fact that many patients might exhibit tumor shrinkage to a degree that did not meet the criteria of RECIST 1.1, but still exhibited significant prolonged survival [10]. Tumor necrosis might occur in response to therapy without appreciable effects on tumor size, despite reduced tumor vascularization and tumor attenuation [10,20,21]. Therefore, response evaluation by RECIST 1.1 may significantly underestimate the number of patients experiencing treatment benefits [7]. Tumor attenuation was previously used to assess treatment response in other cancers [22]. Choi and modified Choi criteria, which included changes in tumor enhancement, were shown to be superior to RECIST 1.1 in their capacity to predict drug efficacy for various cancers [8,9,23,24,25,26,27,28]. Nevertheless, all these assessment tools did not reveal significant differences in Kaplan-Meier curves of the proportion of patients without disease progression, suggesting that existing assessment criteria were not sufficient to reflect underlying alterations in tumor heterogeneity. Therefore, efforts should be made to explore new assessment tools or biomarkers for the identification of pancreatic cancer patients with liver metastasis who might benefit from chemotherapy. Such biomarkers will inform the use of alternative therapies on possible non-responding patients earlier and help better understand disease heterogeneity. 

Heterogeneity of neovascularization reflects conditions of hypoxia inside tumors, which is closely linked to high risk of invasion, metastasis, immunosuppression, and unfavorable response to chemotherapy [29]. Tumor response to a certain treatment could be obviously affected by its heterogenous blood supply. In this case, poor vascularization compromises drug availability at tumor sites. The emerging technique of CT texture analysis permits quantitative extraction of tumor heterogeneity, which enables assessment of tumor neoangiogenesis and hypoxia. Multiple previous studies have characterized the relationship between CT texture parameters and histopathological indicators of hypoxia or angiogenesis in various cancers [30,31]. CT texture analysis may also predict survival and response to treatment in cancer patients [10,32]. To our knowledge, the use of texture parameters as tumor response assessment criteria and predictive biomarkers in pancreatic cancer patients with liver metastasis undergoing chemotherapy has not been explored before.

Our results demonstrated the independent predictive significance of percentage change in liver metastatic lesion SD for TTP in pancreatic cancer patients with liver metastasis. The identified threshold values found by the training cohort were validated by another independent cohort, which increased the generalizability of the present findings [33]. SD is a measure of data dispersion from the mean and it increases in proportion to the square root of the number of objects highlighted and their mean intensity difference relative to background (i.e., dark and bright objects are both positive) [34]. Different from RECIST 1.1, Choi, and modified Choi criteria, the percentage change in texture SD (SSF = 2) of liver metastatic lesions was an independent predictor of TTP. By applying a threshold of −24% for SD at a fine scale (SSF = 2), the performance of the obtained Kaplan-Meier curves of proportion of patients without disease progression was notably different from and better than those obtained by RECIST 1.1, Choi, and modified Choi criteria. This revealed that SD as one of the indicators of tumor heterogeneity provided more details in the level of pixels or gray scales other than traditional enhancement and size change, thereby enabling complementary evaluation. This observation was supported by multiple recent studies involving unresectable pancreatic cancer and pancreatic head cancer [28]. Heterogeneity at fine texture scale was proposed to reflect lesion vascular permeability [35], which suggested that changes in vascular permeability caused by chemotherapy might explain the survival outcomes of pancreatic cancer patients with liver metastasis we observed in this study.

Due to its retrospective nature, this study was constrained by the limited number of participants, thus a prospective study with lager population needed to be conducted in the future to verify our conclusion. Therefore, it was still uncertain that CT texture of liver metastases could modify the clinical management of such patients, especially when multiple liver lesions or large, necrotic lesions presented. Ahn et al. [15] conducted a study to determine whether CT texture analysis of liver metastasis from colorectal cancer is predictive of therapeutic response after cytotoxic chemotherapy, and they found that the lower skewness and narrower SD showed good performance. Nakanishi et al. [36] also conducted a study focusing on the texture analsyis of liver metastasis, and they aimed to develop a radiomics-based prediction model for the response of colorectal liver metastases to oxaliplatin-based chemotherapy. Overall, 126 liver metastases were analyzed and multivariate analysis revealed that high radiomics scores was independently associated with good response. These above-mentioned studies might give us some inspiration for CT texture of liver metastases in response evaluation of pancreatic cancer. Another limitation was that only the largest cross-sectional area of the lesions, not the whole lesions, were analyzed, which therefore might not reflect the characteristics of liver metastatic lesions comprehensively. A 3D texture analysis software should be applied to evaluate the whole lesion heterogeneity and thus reproducibility of the study might be improved. Finally, selection bias could not be avoided as infiltrative metastatic lesions whose boundaries could not be clearly defined, were excluded from analysis. 

## 5. Conclusions

Our findings indicated that the existing assessment criteria, including RECIST 1.1, Choi and modified Choi, were not sufficient for the evaluation of tumor response to treatment. We found that the percentage change in texture SD of liver metastatic lesions derived from contrast-enhanced CT texture analysis, might better predict tumor response and TTP in pancreatic patients with liver metastasis undergoing chemotherapy. Therefore, CT texture was proved to be an effective assessment tool and biomarker that predicted tumor response and TTP in a manner that was superior to traditional response criteria based on enhancement change, size change, or both.

## Figures and Tables

**Table 1:** Main baseline demographics and clinical characteristics of patients in the cohort.

Characteristic	Training Cohort	Validation Cohort	*p* Value
Age (y)	61.1 ± 8.6 (42–76) ^1^	56.1 ± 10.3 (31–73) ^1^	0.055
Gender			0.765
Male	36 (60)	28 (56)	
Female	24 (40)	22 (44)	
CA 19–9 (U/mL)	647.0 (3430.2) {1.2 to 103,641.0} ^2^	778.4 (1589.6) {0.6 to 23,661.0} ^2^	0.14
ECOG Performance Status			0.959
0	38 (63)	32 (64)	
1	22 (37)	18 (36)	
BMI (kg/m^2^)	22.2 ± 2.5 (17.3–28.7) ^1^	22.3 ± 3.4 (18.6–35.5) ^1^	0.998
Number of target lesions			0.707
1	40 (67)	30 (60)	
2	10 (17)	6 (12)	
3	4 (7)	2 (4)	
4	4 (7)	6 (12)	
≥5	2 (3)	6 (12)	
Median time to progression (days)	241 (260) {43 to 1164} ^2^	178 (206) {36 to 1441} ^2^	0.27

Note. Unless otherwise indicated, data are number of patients, with percentages in parentheses. Bold means that the *p* value lower than 0.05 is statistically significant. ^1^ Data are mean ± standard deviation, with range in parentheses for normally distributed data. ^2^ Data are median with interquartile range in parentheses and minimum and maximum in braces for skewed data.

**Table 2:** Response categorization of patients after two treatment cycles.

Response Criteria	Partial Response	Stable Disease	Progressive Disease
RECIST 1.1	16 (27)	36 (60)	8 (13)
Choi	42 (70)	6 (10)	12 (20)
Modified Choi	8 (13)	40 (67)	12 (20)

Note. Data are numbers of patients. Data in parentheses are percentages.

**Table 3:** Median value and percentage change for texture parameters for spatial scaling factor (SSF) at baseline and after two treatment cycles.

		SSF = 0	SSF = 2	SSF = 3	SSF = 4	SSF = 5	SSF = 6
Entropy	Baseline	3.87 (3.01 to 4.45)	4.57 (3.68 to 5.18)	4.61 (3.78 to 5.37)	4.64 (3.38 to 5.54)	4.77 (2.99 to 5.73)	4.85 (2.62 to 5.89)
	After Two Treatment Cycles	3.94 (2.46 to 4.36)	4.62 (2.56 to 5.18)	4.59 (2.56 to 5.37)	4.69 (2.56 to 5.49)	4.72 (2.56 to 5.60)	4.73 (2.56 to 5.70)
	Change (%)	1.54 (−32.60 to 16.57)	0.29 (−32.98 to 11.96)	−0.15 (−34.02 to 20.11)	−0.49 (−34.86 to 39.35)	−1.22 (−34.86 to 56.86)	−1.53 (−34.02 to 73.28)
Mean intensity	Baseline	51.33 (−0.34 to 95.51)	−8.11 (−71.55 to 1438.28)	−18.05 (−99.29 to 2450.36)	−21.23 (−109.29 to 2869.70)	−23.40 (−114.75 to 2762.44)	−17.35 (−176.72 to 2416.81)
	After Two Treatment Cycles	51.45 (−33.42 to 96.56)	−7.94 (−60.70 to 1575.50)	−10.50 (−82.04 to 2823.12)	−13.19 (−110.35 to 3050.04)	−16.25 (−120.03 to 2680.73)	−20.46 (−105.19 to 2166.58)
	Change (%)	2.01 (−72.51 to 9729.41)	22.94 (−487.14 to 8807.41)	−26.59 (−924.15 to 2780.51)	−23.37 (−642.47 to 873.82)	−16.46 (−708.74 to 894.86)	−22.06 (−2135.06 to 1851.01)
Standard deviation	Baseline	15.00 (6.78 to 27.15)	36.56 (19.36 to 211.51)	40.15 (13.39 to 339.41)	45.44 (9.40 to 439.59)	52.37 (6.01 to 413.91)	69.27 (3.74 to 369.51)
	After Two Treatment Cycles	15.39 (8.59 to 24.02)	39.37 (22.12 to 234.82)	46.44 (11.08 to 363.82)	48.44 (12.68 to 425.43)	51.81 (15.86 to 424.24)	52.95 (16.29 to 376.70)
	Change (%)	5.02 (−66.48 to 72.32)	4.36 (−61.88 to 98.55)	−0.38 (−70.23 to 248.47)	−6.54 (−80.10 to 436.38)	−4.80 (−82.34 to 662.06)	−1.11 (−82.07 to 917.91)
Skewness	Baseline	0.14 (−1.51 to 0.93)	0.15 (−2.78 to 3.81)	0.18 (−2.06 to 2.51)	0.09 (−2.23 to 4.67)	0.13 (−2.13 to 4.50)	0.18 (−1.61 to 3.91)
	After Two Treatment Cycles	0.11 (−0.71 to 1.14)	0.27 (−2.36 to 2.50)	0.26 (−1.07 to 3.50)	0.11 (−0.82 to 2.49)	0.11 (−1.36 to 1.78)	0.31 (−1.19 to 1.57)
	Change (%)	−59.81 (−550.00 to 250.00)	−27.47 (−766.67 to 28200.00)	−61.14 (−2150.00 to 625.00)	−62.57 (−7300.00 to 2050.00)	−21.86 (−266.67 to 3287.50)	−22.59 (−1433.33 to 622.22)
Kurtosis	Baseline	−0.08 (−1.32 to 4.32)	0.12 (−0.78 to 20.57)	−0.17 (−1.10 to 15.07)	−0.46 (−1.53 to 32.18)	−0.63 (−1.47 to 25.52)	−0.59 (−1.23 to 17.82)
	After Two Treatment Cycles	−0.14 (−1.06 to 1.67)	−0.01 (−0.95 to 16.10)	−0.20 (−1.22 to 17.78)	−0.58 (−1.61 to 6.91)	−0.71 (−1.47 to 6.66)	−0.42 (−2.23 to 3.41)
	Change (%)	−37.77 (−723.53 to 1566.67)	−75.18 (−700.00 to 2800.00)	−95.27 (−1133.33 to 466.24)	−75.52 (−6350.00 to 1081.25)	−9.03 (−937.11 to 1257.14)	−19.51 (−700.00 to 1566.67)
Mean of positive pixels	Baseline	51.46 (23.50 to 95.51)	24.51 (8.95 to 1438.28)	22.54 (0.00 to 2450.36)	25.58 (0.00 to 2869.70)	28.74 (0.00 to 2762.44)	34.58 (0.00 to 2416.81)
	After Two Treatment Cycles	51.50 (0.00 to 96.56)	26.14 (15.61 to 1575.50)	28.02 (5.50 to 2823.12)	31.03 (6.50 to 3050.04)	30.15 (10.14 to 2680.73)	37.87 (4.59 to 2166.58)
	Change (%)	1.33 (−100.00 to 68.28)	6.97 (−58.48 to 172.39)	6.39 (−64.31 to 2694.62)	4.84 (−73.93 to 4725.30)	2.86 (−84.20 to 3200.00)	6.14 (−82.33 to 7169.00)

Note. Data are median with minimum and maximum in parentheses.

**Table 4:** ROC and Kaplan-Meier analysis for baseline texture parameters at different scale values.

		SSF = 0	SSF = 2	SSF = 3	SSF = 4	SSF = 5	SSF = 6
Entropy	ROC Threshold	>3.52	>4.52	>4.64	>4.88	>4.93	>4.89
	*p* Value	0.55	0.60	0.20	0.28	0.27	0.10
Mean intensity	ROC Threshold	≤52.10	>−9.49	>−18.05	>−19.54	>34.04	≤−47.99
	*p* Value	0.32	**0.04**	0.05	0.18	0.64	0.16
Standard deviation	ROC Threshold	>14.73	>27.58	>58.87	>78.42	>37.47	>39.07
	*p* Value	0.57	0.73	0.15	0.35	0.59	0.49
Skewness	ROC Threshold	>0.32	>0.15	>0.40	>0.45	≤0.03	≤0.37
	*p* Value	0.61	0.43	0.72	0.05	0.22	0.76
Kurtosis	ROC Threshold	>−0.06	>0.01	>0.10	>−0.14	≤−0.97	≤−0.95
	*p* Value	0.61	0.99	**0.03**	**0.04**	0.23	0.13
Mean of positive pixels	ROC Threshold	≤52.54	>27.09	>29.90	>21.10	>16.68	>21.06
	*p* Value	0.32	0.48	0.68	0.24	0.09	0.06

Note. *p* values were obtained with Kaplan-Meier analysis.

**Table 5:** ROC and Kaplan-Meier analysis for texture parameters after two treatment cycles at different scale values.

		SSF = 0	SSF = 2	SSF = 3	SSF = 4	SSF = 5	SSF = 6
Entropy	ROC Threshold	>4.13	>4.93	>4.63	>4.92	>4.97	>4.99
	*p* Value	0.84	0.11	0.66	0.49	0.92	0.92
Mean intensity	ROC Threshold	≤54.58	>−14.82	>−10.50	>−12.75	>−38.76	>−35.90
	*p* Value	0.06	0.59	0.08	0.06	0.78	0.25
Standard deviation	ROC Threshold	≤14.09	>39.37	>49.97	>67.01	>33.73	>39.83
	*p* Value	0.10	0.78	0.84	0.38	0.70	0.65
Skewness	ROC Threshold	≤0.03	≤0.44	≤−0.17	≤−0.13	>−0.46	>−0.47
	*p* Value	0.79	0.56	0.06	0.11	0.70	0.30
Kurtosis	ROC Threshold	>−0.35	>0.64	>−0.29	≤−0.59	≤−0.80	>−0.38
	*p* Value	0.62	0.21	0.53	0.62	0.36	0.22
Mean of positive pixels	ROC Threshold	≤54.58	>25.63	>104.25	>33.97	>30.15	>12.15
	*p* Value	0.06	0.31	0.50	0.47	0.33	0.70

Note. *p* values were obtained with Kaplan-Meier analysis.

**Table 6:** ROC and Kaplan-Meier analysis for percentage change in texture parameters at different scale values.

		SSF = 0	SSF = 2	SSF = 3	SSF = 4	SSF = 5	SSF = 6
Entropy	ROC Threshold	≤4.53	≤0.66	≤0.97	≤3.34	≤0.51	≤8.85
	*p* Value	0.07	0.08	0.09	0.36	**0.03**	0.48
Mean intensity	ROC Threshold	>0.19	≤−119.23	>−8.32	>−73.13	>−91.35	>−82.74
	*p* Value	0.85	0.06	0.33	0.44	0.87	0.55
Standard deviation	ROC Threshold	≤48.66	≤−23.61	≤75.13	≤4.30	≤8.05	≤31.01
	*p* Value	0.16	**0.02**	0.34	0.26	0.22	0.28
Skewness	ROC Threshold	>−59.81	≤35.98	≤94.54	>−87.63	>−37.60	≤−679.84
	*p* Value	0.26	0.54	0.34	0.17	0.62	0.95
Kurtosis	ROC Threshold	>−97.41	>−112.04	>−2.98	>13.24	>−32.10	≤−95.41
	*p* Value	0.19	**0.02**	0.89	0.33	0.73	0.86
Mean of positive pixels	ROC Threshold	>0.19	≤6.65	≤3.85	≤2.62	≤2.86	≤12.84
	*p* Value	0.80	0.10	0.67	**0.02**	**0.005**	0.22

Note. *p* values were obtained with Kaplan-Meier analysis.

**Table 7:** Analysis of survival with multivariate cox regression model.

Parameter	Multivariate	
	HR	*p* Value
Baseline (SSF = 2)		
Age	1.00 (0.93, 1.07)	1.00
Gender	1.13 (0.43, 2.96)	0.80
CA19-9	0.20 (0.02, 1.63)	0.13
BMI	0.99 (0.73, 1.33)	0.94
Mean intensity	0.10 (0.01, 0.78)	**0.028**
Baseline (SSF = 3)		
Age	0.99 (0.93, 1.06)	0.84
Gender	0.85 (0.32, 2.26)	0.74
CA19-9	1.26 (0.41, 3.85)	0.69
BMI	1.06 (0.83, 1.34)	0.66
Kurtosis	0.33 (0.12, 0.91)	**0.032**
Baseline (SSF = 4)		
Age	0.99 (0.93, 1.06)	0.85
Gender	0.73 (0.27, 1.95)	0.53
CA19-9	1.24 (0.39, 3.88)	0.72
BMI	1.02 (0.79, 1.31)	0.88
Kurtosis	0.36 (0.13, 1.00)	**0.049**
Percentage change (SSF = 2)		
Age	0.98 (0.91, 1.06)	0.61
Gender	1.80 (0.59, 5.46)	0.30
CA19-9	1.12 (0.36, 3.51)	0.85
BMI	0.99 (0.80, 1.24)	0.96
Standard deviation	3.62 (1.70, 9.87)	**0.016**
Kurtosis	0.24 (0.06, 0.89)	**0.033**
Percentage change (SSF = 4)		
Age	1.00 (0.93, 1.07)	0.90
Gender	0.70 (0.26, 1.88)	0.48
CA19-9	1.68 (0.56, 5.07)	0.36
BMI	1.01 (0.79, 1.30)	0.91
Mean of positive pixels	3.45 (1.27, 9.41)	**0.016**
Percentage change (SSF = 5)		
Age	1.00 (0.93, 1.07)	0.89
Gender	0.68 (0.24, 1.93)	0.47
CA19-9	1.79 (0.56, 5.76)	0.33
BMI	1.02 (0.80, 1.30)	0.88
Entropy	0.99 (0.21, 4.69)	0.99
Mean of positive pixels	4.55 (0.97, 21.48)	0.06

Note. Data in parentheses are 95% confidence intervals. Bold means that the *p* value lower than 0.05 is statistically significant.

**Table 8:** Univariate Kaplan-Meier analysis of texture parameters for predicting survival in the validation cohort.

Parameter	Value	Median Time to Progression (d)		*p* Value
		Above Optimal Threshold	Below Optimal Threshold	
Baseline				
Mean intensity (SSF = 2)	−6.95 [−53.76 to 466.36]	179 (14)	204 (11)	0.32
Kurtosis (SSF = 3)	0.12 [−1.14 to 29.18]	177 (15)	242 (10)	0.37
Kurtosis (SSF = 4)	0.31 [−1.15 to 30.21]	204 (14)	175 (11)	0.29
Percentage change (%)				
Entropy (SSF = 5)	0.10 [−16.80 to 19.08]	177 (10)	204 (15)	0.97
Standard deviation (SSF = 2)	−0.51 [−76.00 to 72.79]	242 (17)	59 (8)	**0.019**
Kurtosis (SSF = 2)	−72.50 [−2320.00 to 781.82]	177 (18)	204 (7)	0.35
Mean of positive pixels (SSF = 4)	−8.67 [−65.93 to 321.14]	179 (9)	204 (16)	0.90
Mean of positive pixels (SSF = 5)	−8.82 [−82.09 to 310.41]	179 (9)	204 (16)	0.75

Note. Data are median with minimum and maximum in brackets and numbers in parentheses are numbers of patients. Bold means that the *p* value lower than 0.05 is statistically significant.

## Data Availability

The data presented in this study are available on request from the corresponding author. The data are not publicly available due to privacy.
